# Similarities and Differences in the Immune Characteristics of Intestinal Gamma Delta T Cells From Patients With Crohn's Disease and Ulcerative Colitis and Their Correlation With Disease Activity

**DOI:** 10.1002/iid3.70273

**Published:** 2025-10-15

**Authors:** Yujie Jiang, Linna Ye, Caixia Sheng, Jia Zhu, Jiaqi Xu, Xiaoqing Cheng, Guoxiang Fu, Zhinong Jiang

**Affiliations:** ^1^ Department of Pathology Sir Run Run Shaw Hospital Affiliated With Zhejiang University School of Medicine Hangzhou China; ^2^ Department of Gastroenterology Sir Run Run Shaw Hospital Affiliated With Zhejiang University School of Medicine Hangzhou China

**Keywords:** Crohn's disease, disease activity, gamma delta T cell, immune biomarker, ulcerative colitis

## Abstract

**Background:**

Intestinal γδ T‐cell immune characteristics and their relationship with disease activity in Crohn's disease (CD) and ulcerative colitis (UC) remain to be fully clarified.

**Methods:**

Biopsies from 21 CD, 21 UC and 21 healthy controls were analyzed by flow cytometry for γδ T‐cell frequency, cytotoxicity (perforin, granzyme‐B), activation (HLA‐DR) and exhaustion (PD‐1). ROC curves were used to evaluate the diagnostic performance of these indices. Vγ subsets were profiled using published scRNA‐seq data.

**Results:**

As disease activity increased, intestinal γδ T cells in CD and UC patients decreased and could not activate. The differences were that the cytotoxicity of intestinal γδ T cells in CD patients was always normal as disease activity increased. In contrast, the cytotoxicity of intestinal γδ T cells in UC patients was suppressed. Different Vγ subsets in CD or UC patients showed different immune characteristics, which might lead to different immune characteristics of γδT cells in CD or UC patients at different disease active stages. Furthermore, γδ T cell and HLA‐DR+ γδ T cell ratio were good indicators in diagnosing CD. The ratios of γδ T cell, HLA‐DR+ γδ T cell, PD‐1+ γδ T cell, and Perforin+ γδ T cell exhibited values for diagnosing UC. PD‐1+ γδ T cell ratio was a valuable indicator to help distinguish CD from UC.

**Conclusion:**

Intestinal γδ T cells exhibit both shared and divergent features in CD and UC that closely parallel disease activity, supporting their potential as immune biomarkers for diagnosis and discrimination between the two diseases.

## Graphical abstract

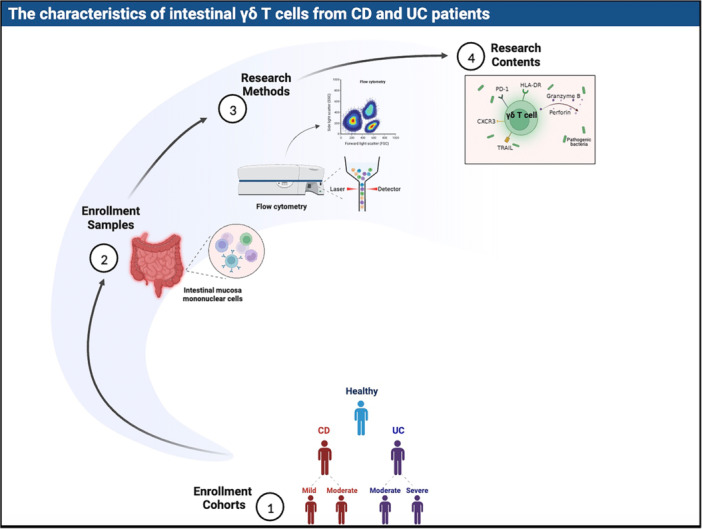

## 1 Introduction

Inflammatory bowel disease (IBD) is a chronic intestinal inflammation, which includes Crohn's disease (CD) and ulcerative colitis (UC) [1, 2]. IBD was once most frequent in Europe and North America and less common in non‐Western countries. However, the incidence rates in Asia have climbed rapidly in recent decades. IBD contributed to an ever‐increasing economic burden on healthcare systems and significantly reduced people's quality of life [3, 4, 5]. The etiology of IBD is caused by susceptibility gene variants, environmental changes, gut microbiome imbalances, and dysregulated immune responses. IBD is a chronic intestinal inflammation that is ultimately a dysregulated immune response. Therefore, current research on the pathogenesis of IBD mainly focuses on immune abnormalities. T lymphocytes can be classified into two subgroups based on TCR expression: alpha‐beta T (αβ T) cells and gamma‐delta T (γδ T) cells [6]. The importance of the conventional T cells (αβ T) in IBD is now generally recognized, but the role of the γδ T cells in IBD remains to be further investigated.

Unconventional γδ T cells differ in biology and function from αβ T cells. Since γδ T cells lack the major histocompatibility complex (MHC) restriction, they can rapidly recognize microorganisms, infect or transform host cells, and exert direct cytotoxic effects by secreting granzyme and perforin [7, 8]. γδ T cells are critical in the first line of defense against infection and intestinal wound healing [9, 10, 11, 12]. They constitute only a small population of T lymphocytes in the periphery but are enriched in the epithelium, particularly abundant in the intestine [13]. Existing studies had mainly focused on the impact of γδ T cells in a single disease (CD or UC) and the combination of CD and UC patients into IBD patients to research γδ T cells in them [14, 15]. Moreover, the relationship between the immune characteristics of γδ T cells and disease activity in CD and UC patients is unclear. We have previously studied the immune characteristics of CD8+ γδ T cell subsets in CD patients, and obtained some interesting findings [16]. However, we did not study the immune characteristics of γδ T cells in patients with CD and UC, and compared them together. Therefore, we carried out this study. In this study, intestinal mucosa was obtained from CD and UC patients and healthy controls. First, we examined γδ T cell distribution and immunological function in CD and UC patients' intestinal mucosa. Subsequently, we further explored the immune characteristics of γδ T cells in the intestinal mucosa of CD and UC patients with varying degrees of disease activity. Through the study, we have a more comprehensive understanding of the similarities and differences in intestinal γδ T cells' immune characteristics from CD and UC patients and their correlation with disease activity. Furthermore, by analyzing the existing single cell RNA sequencing (ScRNA‐seq) data [17], we found that the different Vγ subsets in CD or UC patients show different immune characteristics, which may also contribute to the differences in immune characteristics of γδT cells in CD or UC patients at different disease active stages. In addition, we found that the phenotypic markers of CD‐or‐UC specific perturbations in intestinal γδ T cells could be used as indicators to assist in the diagnosis of CD or UC and to distinguish CD from UC, which might become potential immune biomarkers.

## 2 Materials and Methods

### 2.1 Characteristics of Sample Cohort

#### 2.1.1 Human Intestinal Biopsy Samples

The study analyzed fresh intestinal biopsy samples from healthy controls (*n* = 21), CD patients (*n* = 21), and UC patients (*n* = 21). CD and UC patients were diagnosed by endoscopy, histological criteria, radiological studies, and clinical parameters. All enrolled patients were required to be free from cancer, other autoimmune diseases, and infectious diseases like hepatitis B or tuberculosis. They should also not have had intestinal surgery, blood transfusion, organ or bone marrow transplantation, or immunosuppressive drugs in the past year. Only patients in the active phase were included, while those in remission were excluded. Clinically, the Crohn's Disease Activity Index (CDAI) and Ulcerative Colitis Disease Activity Index (UCDAI) were used to evaluate the CD and UC disease activity. A CDAI score of < 150 indicates disease remission, 150–220 indicates mild activity, 220–450 indicates moderate activity, and > 450 indicates severe activity. A UCDAI score of ≤ 2 indicates disease remission, 3–5 indicates mild activity, 6–10 indicates moderate activity, and 11–12 indicates severe activity. This study categorized CD patients with intestinal biopsy samples into mild (*n* = 10) and moderate (*n* = 11) activity. UC patients with intestinal biopsy samples were categorized into moderate (*n* = 13) and severe (*n* = 8) activity. In this study, intestinal biopsy samples of the enrolled cohorts were all obtained from the colonic mucosa. Intestinal biopsy samples from CD and UC patients were obtained from colonic mucosa that was macroscopically inflamed but non‐ulcerated. Intestinal biopsy samples of healthy controls were obtained from the colonic mucosa of subjects who underwent endoscopy but exhibited no endoscopic abnormalities. Intestinal biopsy samples required for the study were obtained from the Department of Gastroenterology, Sir Run Run Shaw Hospital, Affiliated with Zhejiang University School of Medicine, Hangzhou, China. The study received authorization from the ethics committee of Sir Run Run Shaw Hospital, Affiliated with Zhejiang University School of Medicine (number: 2022‐0293). The study followed the Declaration of Helsinki principles. All subjects had signed written informed consents. No significant differences were determined among study cohorts for the age and gender ratio. The clinical characteristics of the tissue donors are summarized in Table 1.

**Table 1:** Clinical characteristics of the tissue donors in the study.

Group	HC	CD	UC
Case	21	21	21
Sex (male/female)	12/9	14/7	16/5
Age (years)	43 (16–73)	26 (15–61)	47 (15–74)
CDAI score (< 150/150–220/220–450/ > 450)	ND	0/10/11/0	ND
UCDAI score (≤ 2/3–5/6–10/11–12)	ND	ND	0/0/13/8
Disease activity (Remission/Mild/Moderate/Severe)	ND	0/10/11/0	0/0/13/8

*Note:* Data are shown as median and range.

Abbreviations: > 450, severe activity; 11–12, severe activity; 150–220, mild activity; 220–450, moderate activity; 3–5, mild activity; 6–10, moderate activity; CD, Crohn's disease; CDAI score < 150, disease remission; CDAI, Crohn's Disease Activity Index; HC, healthy control; ND, not determined; UC, ulcerative colitis; UCDAI score ≤ 2, disease remission; UCDAI, Ulcerative Colitis Disease Activity Index.

### 2.2 Sample Acquisition and Processing

Intestinal mucosal tissues from IBD patients and HCs were freshly obtained during endoscopy. The collected tissues were preserved in sterile PBS and promptly transported to the laboratory for further processing. Initially, the intestinal mucosal tissues were finely minced into small pieces. After incubation in Hank's Balanced Salt Solution containing EDTA and DTT for 40 min, tissues were digested with Collagenase type IV and DNase for 2 h on a shaker at 37°C. The digested cell suspension was filtered through a 70‐μM nylon mesh, and the filtrate was washed twice in sterile PBS.

### 2.3 Fluorescence‐Activated Flow Cytometry Analysis

We utilized cells derived from the intestinal mucosa to assess the phenotypic and functional characteristics of γδ T cells. The cells were subjected to cell surface staining, fixation, and permeabilization to enable intracellular staining. Cell staining was performed with the following fluorochrome‐conjugated antibodies: Zombie Red Fixable Viability stain (BioLegend); Brilliant Violet 510 anti‐human CD45 mAb (Biolegend, USA), APC/Fire 750 anti‐human CD8 mAb (Biolegend, USA), PerCP/Cyanine5.5 anti‐human CD56 (NCAM) mAb (Biolegend, USA), FITC anti‐human CD3 mAb (Biolegend, USA), Alexa Fluor 700 anti‐human/mouse Granzyme B Recombinant mAb (Biolegend, USA), APC anti‐human TCR Vα7.2 mAb (Biolegend, USA), Brilliant Violet 650 anti‐human CD183 (CXCR3) mAb (Biolegend, USA), PE anti‐human CD279 (PD‐1) mAb (Biolegend, USA), PE anti‐human Perforin mAb (Biolegend, USA), Brilliant Violet 785 anti‐human HLA‐DR mAb (Biolegend, USA), Brilliant Violet 421 anti‐human TCR γ/δ mAb (Biolegend, USA). Cell fluorescence intensity was determined via a DxFLEX flow cytometer (Beckman, USA). At least 100,000 events were acquired for each sample. Flow cytometry results were analyzed by the FlowJo software (Tree Star). The gating strategy of lymphocytes, αβ T cells, and γδ T cells was executed as CD45+, CD45 + CD3 + TCR γ/δ −, CD45 + CD3 + TCR γ/δ+, respectively. To account for the continuous expression pattern of fluorescent markers on the mentioned partial antibodies (CXCR3, HLA‐DR, PD‐1, Perforin, and Granzyme B), we employed Fluorescence Minus One (FMO) staining. The FMO staining results are included in the Supporting Information S1: Figure A1.

### 2.4 Analysis of ScRNA‐Seq Data

Publicly available scRNAseq data set (GSE214695) Data from Garrido‐Trigo, Alba et al. were downloaded and reanalyzed by us [17]. This data set includes three groups of colon samples (*n* = 18 samples) including active Crohn's disease (CD, *n* = 6), active ulcerative colitis (UC, *n* = 6) and healthy control group (HC, *n* = 6). The data set underwent standardization procedures, including quality control, background correction, and normalization to ensure consistency and comparability in our analysis. We calculated the average expression levels of each cluster in the data set at single cell level.

### 2.5 Statistical Analysis

GraphPad Prism software (v.9.4.0 Inc. San Diego, CA, USA) was used for statistical analysis. We computed Mann–Whitney U tests for single comparisons, and we used two‐way ANOVAs for multiple comparisons. The diagnostic value of each indicator was appraised using the receiver operation characteristic (ROC) curves. The 95% confidence interval was utilized to calculate the sensitivity, specificity and consistency, and the cut‐off value was selected when the Jordan index was at its maximum. In the text, we described frequency as means unless stated otherwise. The data on the graphs were expressed as mean ± SEM. A two‐sided *p* < 0.05 was considered significant for all tests. The statistical significance was indicated as follows: ****p < 0.0001, ***p < 0.001, **p < 0.01, and *p < 0.05. Not significant: ns; *p* > 0.05.

## 3 Results

### 3.1 Distribution of Intestinal γδ T Cells in CD and UC Patients

**Figure 1:**
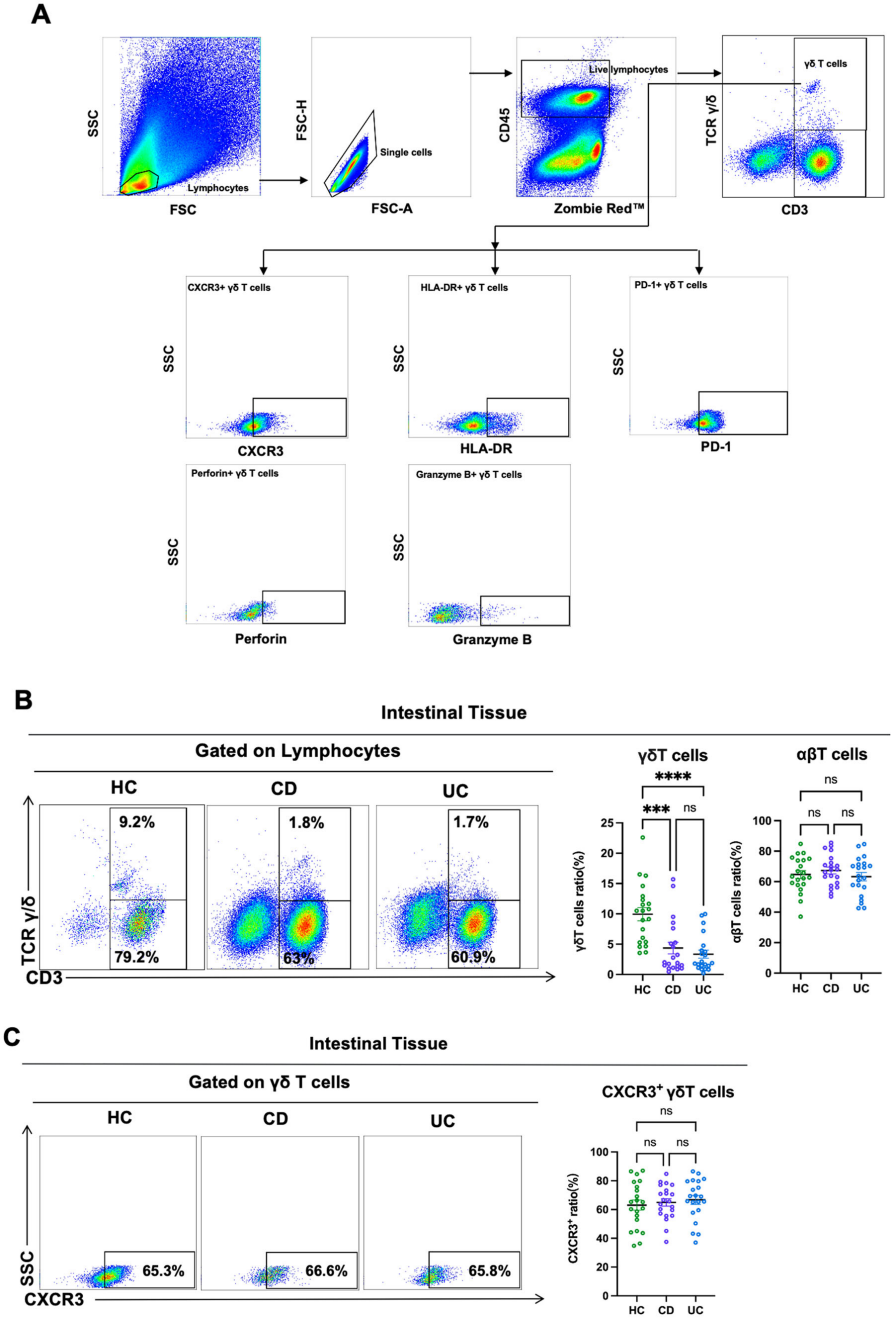
Distribution of intestinal γδ T cells in CD and UC patients. (A) A full gating of flow cytometry plots of the enrolled cohorts. (B) Representative dot plots of intestinal γδ T (CD3 + TCR γδ+) and αβ T cells (CD3 + TCR γδ−) in HCs, CD patients, and UC patients were shown. Values in the quadrant represent the percentages of γδ T and αβ T cells among lymphocytes. Pooled data compared the intestinal γδ T and αβ T cell frequency in HCs, CD patients, and UC patients. (C) Representative dot plots of the CXCR3 expression on intestinal γδ T cells in HCs, CD patients, and UC patients were shown. Values in the quadrant represent the percentages of the CXCR3 expression on γδ T cells. Pooled data compared the CXCR3 expression on intestinal γδ T cells in HCs, CD, and UC patients. CD, Crohn's disease; CXCR3, chemokine receptor marker; HCs, healthy controls; UC, ulcerative colitis. Data are mean ± SEM. *p < 0.05; **p < 0.01; ***p < 0.001; ****p < 0.0001.

The distribution of γδ T cells and conventional T cells (αβ T cells) in all enrollees' intestinal mucosa was analyzed by flow cytometry. The gating strategy is shown in Figure 1A. The results showed the frequency of intestinal γδ T cells in CD and UC patients was significantly decreased compared to HCs (Figure 1B). To investigate the decrease in γδ T cells in CD and UC patients' intestinal mucosa, we compared the frequency of conventional T cells (αβ T cells) in the three cohorts' intestinal mucosa. We found no significant difference in intestinal αβ T cells from the three cohorts (Figure 1B). To further explore the reasons for the decrease in intestinal γδ T cells from CD and UC patients, we investigated the chemokine receptor CXCR3 expression on intestinal γδ T cells. This study aimed to assess the migratory potential of γδ T cells. We found that the CXCR3 expression on intestinal γδ T cells had no significant differences among the three groups (Figure 1C). Therefore, we speculated that decreased intestinal γδ T cells in CD and UC patients might be related to increased cell death and reduced cell proliferation, rather than αβ T cells expansion or altered migratory capacity of γδ T cells.

### 3.2 Distribution of Intestinal γδ T Cells in CD and UC Patients With Varying Degrees of Disease Activity

**Figure 2:**
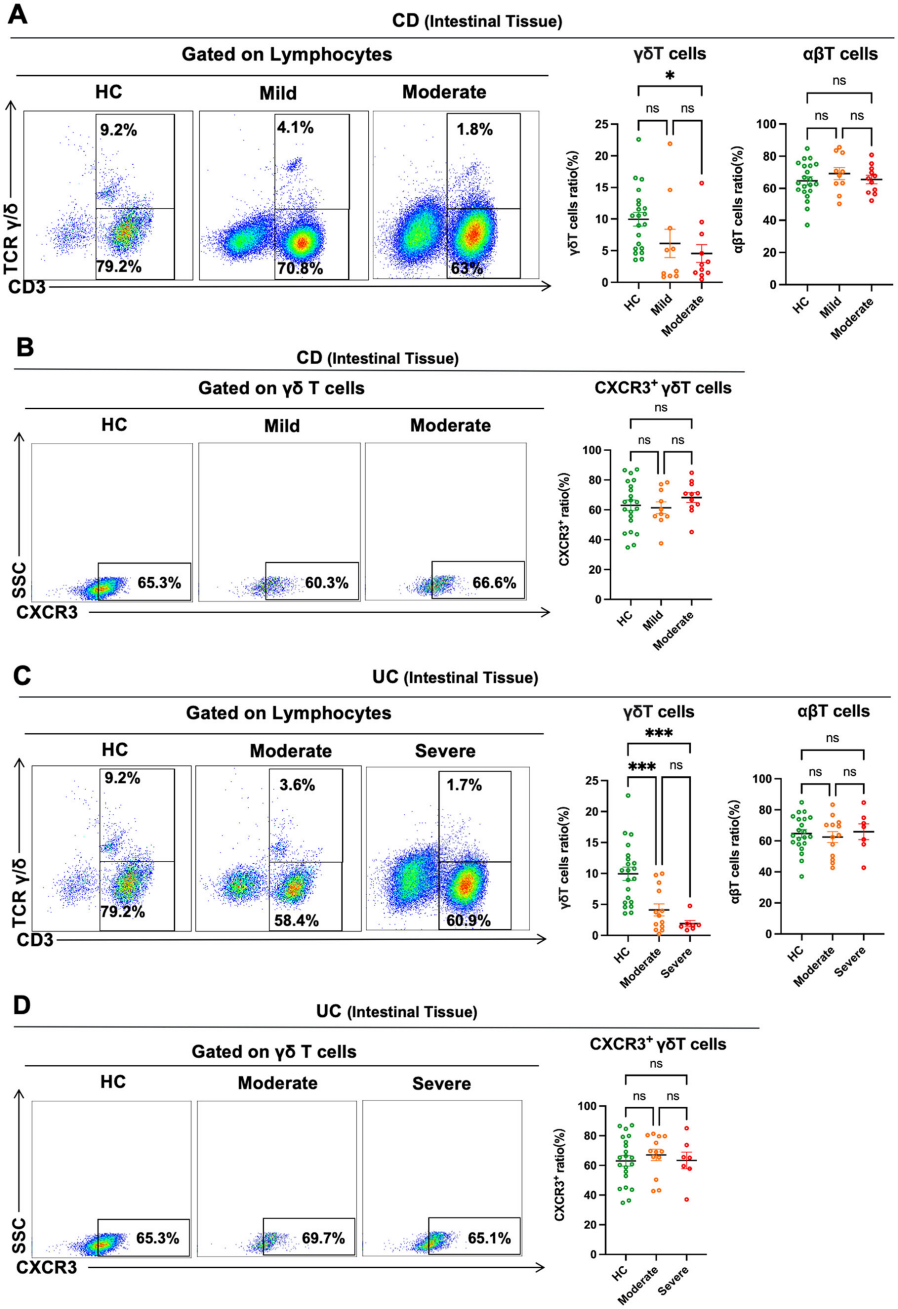
Distribution of intestinal γδ T cells in CD and UC patients with varying degrees of disease activity. (A) Representative dot plots of intestinal γδ T (CD3 + TCR γδ+) and αβ T cells (CD3 + TCR γδ−) in HCs, CD patients with mild activity, CD patients with moderate activity were shown. Values in the quadrant represent the percentages of γδ T and αβ T cells among lymphocytes. Pooled data compared the intestinal γδ T and αβ T cell frequency in HCs, CD patients with mild activity, and CD patients with moderate activity. (B) Representative dot plots of the CXCR3 expression on intestinal γδ T cells in HCs, CD patients with mild activity, and CD patients with moderate activity were shown. Values in the quadrant represent the percentages of the CXCR3 expression on γδ T cells. Pooled data compared the CXCR3 expression on intestinal γδ T cells in HCs, CD patients with mild activity, and CD patients with moderate activity. (C) Representative dot plots of intestinal γδ T (CD3 + TCR γδ + ) and αβ T cells (CD3 + TCR γδ−) in the intestinal mucosa from HCs, UC patients with moderate activity, UC patients with severe activity were shown. Values in the quadrant represent the percentages of γδ T and αβ T cells among lymphocytes. Pooled data compared the γδ T and αβ T cells frequency in the intestinal mucosa from HCs, UC patients with moderate activity, and UC patients with severe activity. (D) Representative dot plots of the CXCR3 expression on intestinal γδ T cells in HCs, UC patients with moderate activity, and UC patients with severe activity were shown. Values in the quadrant represent the percentages of the CXCR3 expression on γδ T cells. Pooled data compared the CXCR3 expression on intestinal γδ T cells in HCs, UC patients with moderate activity, and UC patients with severe activity. Data are mean ± SEM. *p < 0.05; **p < 0.01; ***p < 0.001; ****p < 0.0001.

Further investigation examined the association between intestinal γδ T cell distribution and disease activity in CD and UC patients. First, we examined the intestinal γδ T cell distribution in CD patients with varying degrees of disease activity. The results showed that the frequency of intestinal γδ T cells in CD patients with mild activity was also not substantially different from that of the HCs. However, the frequency of intestinal γδ T cells in CD patients with moderate activity was lower than that of the HCs. Furthermore, we found that intestinal αβ T cell frequency in CD patients with varying degrees of disease activity was comparable to that of the HCs (Figure 2A). Moreover, we investigated that the CXCR3 expression on intestinal γδ T cells in CD patients with varying degrees of disease activity was not significantly different (Figure 2B).

Subsequently, we analyzed intestinal γδ T cell distribution in UC patients with varying degrees of disease activity. Intestinal γδ T cell frequency was considerably reduced in moderate and severe UC patients compared to HCs. Meanwhile, consistent with the results in CD patients with varying degrees of disease activity, we found that the frequency of intestinal αβ T cells in UC patients with varying degrees of disease activity was not substantially different from those of HCs (Figure 2C). UC patients with diverse degrees of disease activity showed no significant difference in CXCR3 expression on intestinal γδ T cells (Figure 2D). From the above results, we found that a reduction of intestinal γδ T cells in CD and UC patients might be related to disease activity.

### 3.3 Immune Function of Intestinal γδ T Cells in CD and UC Patients

**Figure 3:**
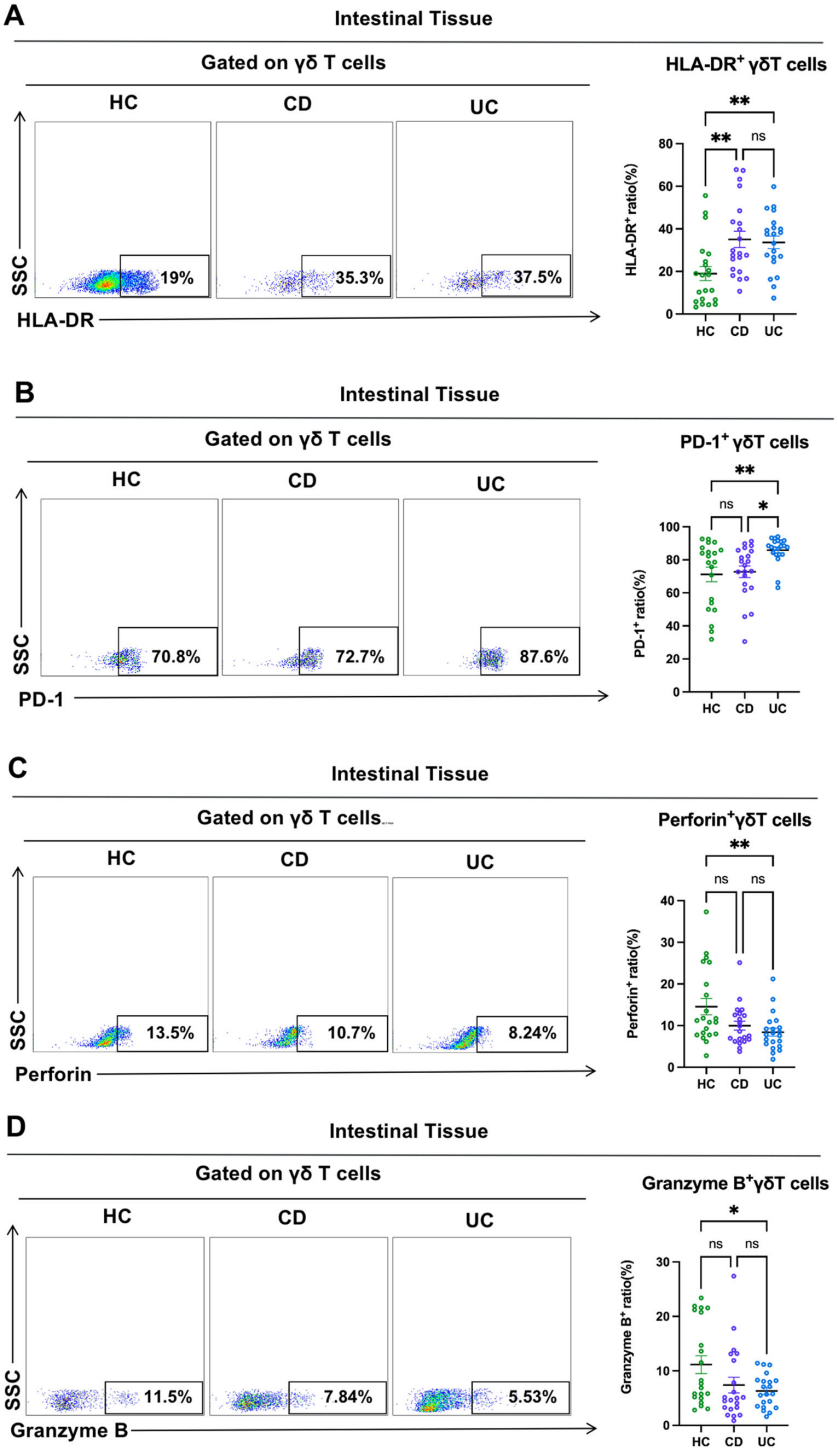
Immune function of intestinal γδ T cells in CD and UC patients. (A–D) Representative dot plots of the markers (HLA‐DR, PD‐1, Perforin, and Granzyme B) expression in intestinal γδ T cells of HCs, CD patients, and UC patients were shown. Values in the quadrant represent the percentages of the markers (HLA‐DR, PD‐1, Perforin, and Granzyme B) expression in γδ T cells. Pooled data compared the markers (HLA‐DR, PD‐1, Perforin, and Granzyme B) expression in intestinal γδ T cells of HCs, CD patients and UC patients. HLA‐DR, activation marker; PD‐1, immunosuppressive molecule, perforin and Granzyme B, cytotoxic molecules. Data are mean ± SEM. *p < 0.05; **p < 0.01; ***p < 0.001; ****p < 0.0001.

To determine if γδ T cells in CD and UC patients had activated or suppressed immune activity, we detected the activation marker HLA‐DR and the immunosuppressive molecule PD‐1. We found the HLA‐DR expression on intestinal γδ T cells was significantly increased in CD and UC patients compared to HCs (Figure 3A). Furthermore, we found that PD‐1 expression on intestinal γδ T cells was significantly increased in UC patients compared to the other two cohorts (Figure 3B). To understand better the immune function of intestinal γδ T cells in CD and UC patients, we examined the cytotoxic molecules (Perforin and Granzyme B) expression in intestinal γδ T cells. The results showed no significant difference in the expression of Perforin and Granzyme B in intestinal γδ T cells of CD patients compared to HCs. However, the expression of cytotoxic molecules (Perforin and Granzyme B) was significantly decreased in intestinal γδ T cells of UC patients compared to HCs (Figure 3C,D). The above results indicated a significant difference in the immune function of intestinal γδT cells between CD and UC patients. We speculated that there might be differences in intestinal γδ T cells' immunological function in CD and UC patients with varying disease activity.

### 3.4 Immune Function of Intestinal γδ T Cells in CD Patients With Varying Degrees of Disease Activity

**Figure 4:**
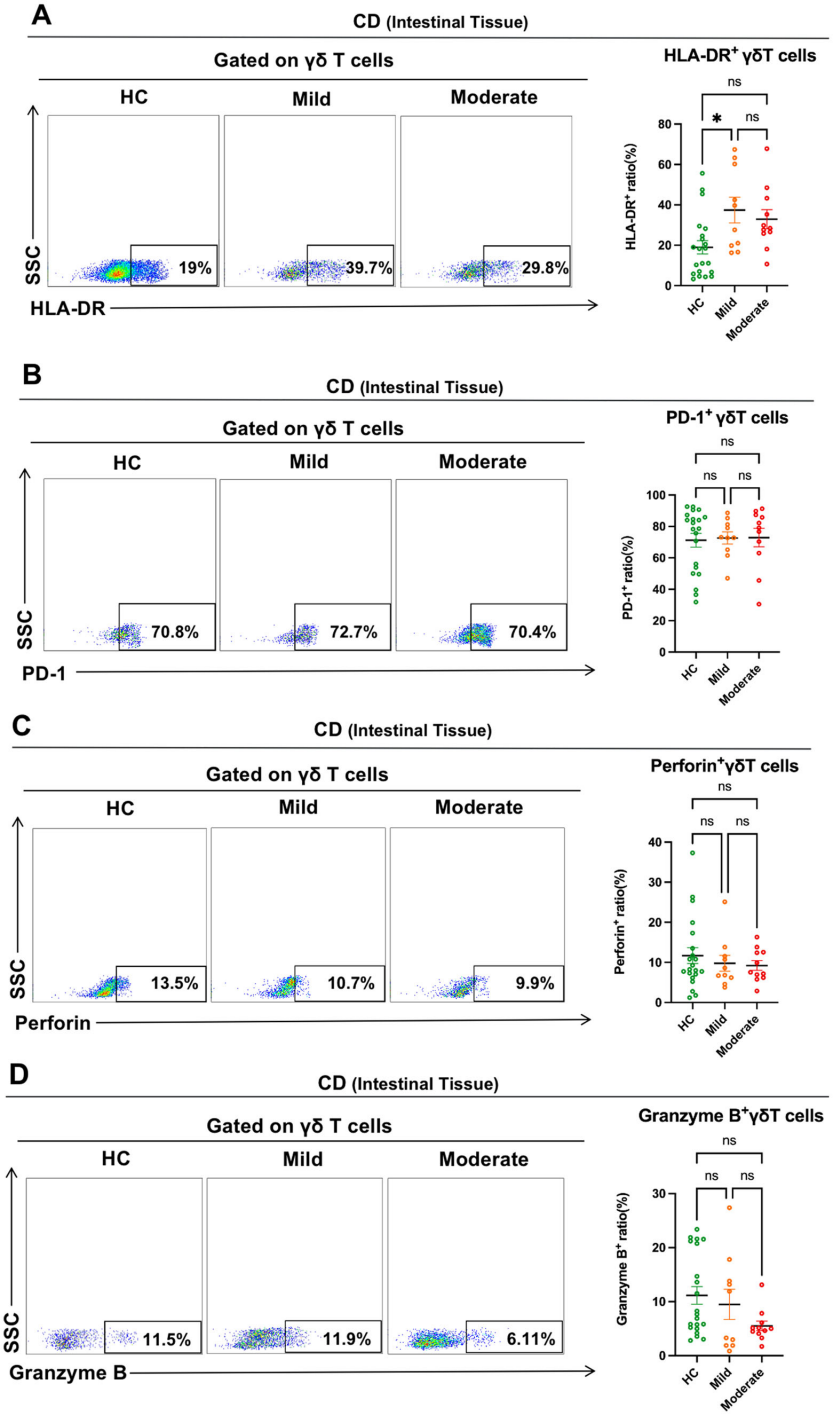
Immune function of intestinal γδ T cells in CD patients with varying degrees of disease activity. (A–D) Representative dot plots of the markers (HLA‐DR, PD‐1, Perforin, and Granzyme B) expression on intestinal γδ T cells in HCs, CD patients with mild activity, and CD patients with moderate activity were shown. Values in the quadrant represent the percentages of the markers (HLA‐DR, PD‐1, Perforin, and Granzyme B) expression on γδ T cells. Pooled data compared the markers (HLA‐DR, PD‐1, Perforin, and Granzyme B) expression on intestinal γδ T cells in HCs, CD patients with mild activity, and CD patients with moderate activity. Data are mean ± SEM. *p < 0.05; **p < 0.01; ***p < 0.001; ****p < 0.0001.

We investigated the immunological function of intestinal γδ T cells in CD and UC patients with varing degrees of disease activity to better understand the relationship between disease severity and their function. First, we investigated the immune function of intestinal γδ T cells in CD patients with varying degrees of disease activity. There was no significant difference in HLA‐DR expression on intestinal γδ T cells between CD patients with moderate activity and HCs. HLA‐DR expression on intestinal γδ T cells in mild active CD patients was higher than in HCs (Figure 4A). We then found that PD‐1 expression on intestinal γδ T cells in CD patients with varying degrees of disease activity did not differ substantially from that of the HCs (Figure 4B). Furthermore, we found that the expression of cytotoxic molecules (Perforin and Granzyme B) in intestinal γδ T cells of CD patients with varying disease activity were not significantly different (Figure 4C,D). From the above results, we concluded that the intestinal γδ T cells in CD patients with mild activity were activated, but as disease activity increased in CD patients, intestinal γδ T cells could not activate in response to inflammatory stimuli, but their cytotoxicity remained unimpaired.

### 3.5 Immune Function of Intestinal γδ T Cells in UC Patients With Varying Degrees of Disease Activity

**Figure 5:**
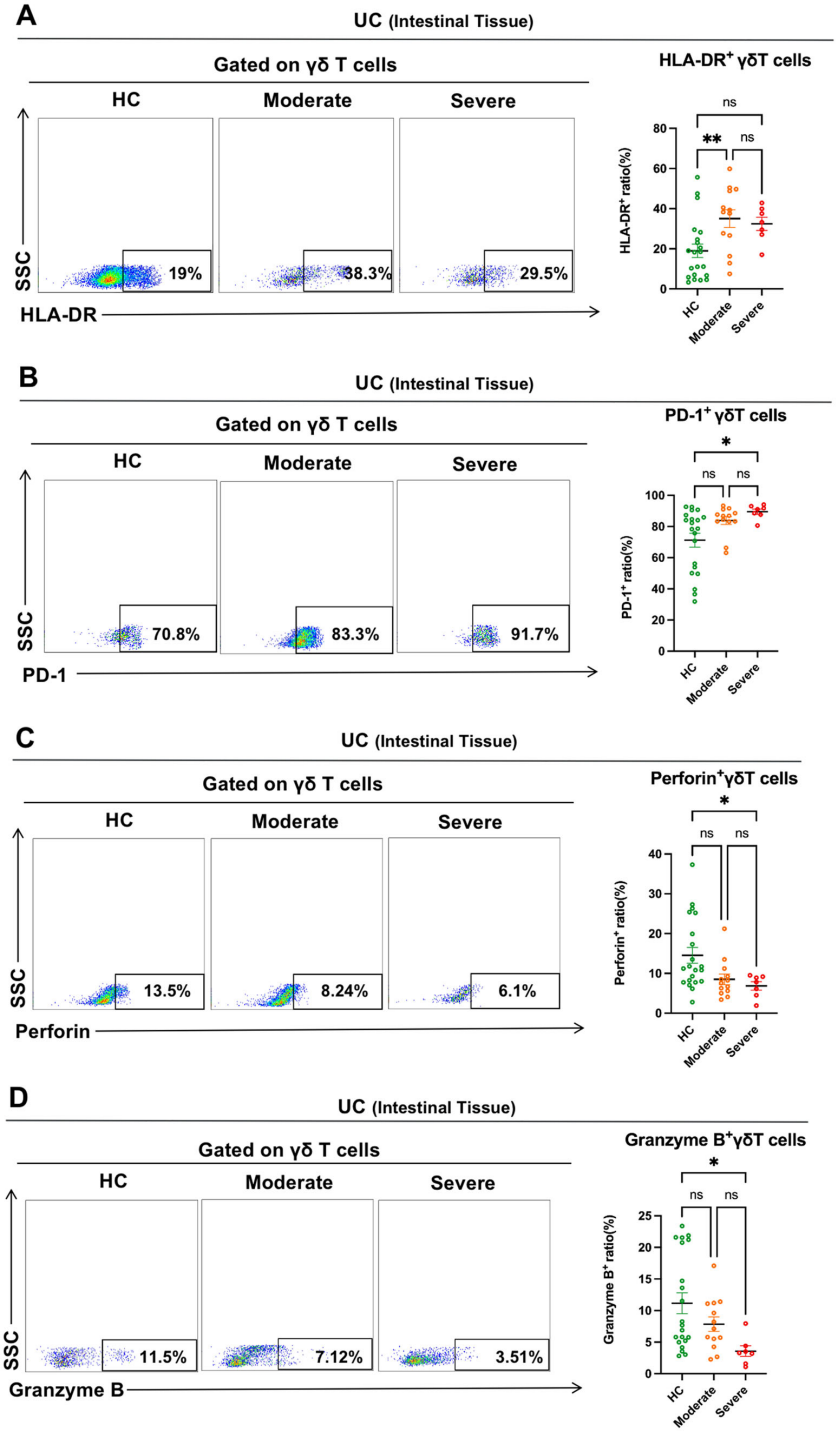
Immune function of intestinal γδ T cells in UC patients with varying degrees of disease activity. (A–D) Representative dot plots of the markers (HLA‐DR, PD‐1, Perforin, and Granzyme B) expression on intestinal γδ T cells in HCs, UC patients with moderate activity, and UC patients with severe activity were shown. Values in the quadrant represent the percentages of the markers (HLA‐DR, PD‐1, Perforin, and Granzyme B) expression on γδ T cells. Pooled data compared the markers (HLA‐DR, PD‐1, Perforin, and Granzyme B) expression on intestinal γδ T cells in HCs, UC patients with moderate activity, and UC patients with severe activity. Data are mean ± SEM. *p < 0.05; **p < 0.01; ***p < 0.001; ****p < 0.0001.

Next, we study the immune function of intestinal γδ T cells in UC patients with various disease activities. Our study found greater HLA‐DR expression on intestinal γδ T cells in moderately active UC patients than HCs. However, the HLA‐DR expression on intestinal γδ T cells in severely active UC patients was not substantially different from that of the HCs (Figure 5A). Moreover, we found that the PD‐1 expression on intestinal γδ T cells in UC patients with severe activity was higher than the HCs (Figure 5B). Furthermore, cytotoxic molecules (Perforin and Granzyme B) expression was considerably reduced in intestinal γδ T cells of UC patients with severe activity compared to HCs (Figure 5C,D). Based on the above results, we concluded that UC patients' intestinal γδ T cells were still activated when the disease activity was not serious. However, with the increase of disease activity, the intestinal γδ T cells in UC patients with severe activity were suppressed with impaired cytotoxicity.

### 3.6 ScRNA‐Seq Analysis of Intestinal γδ T Cells and Their Subsets in CD and UC Patients

**Figure 6:**
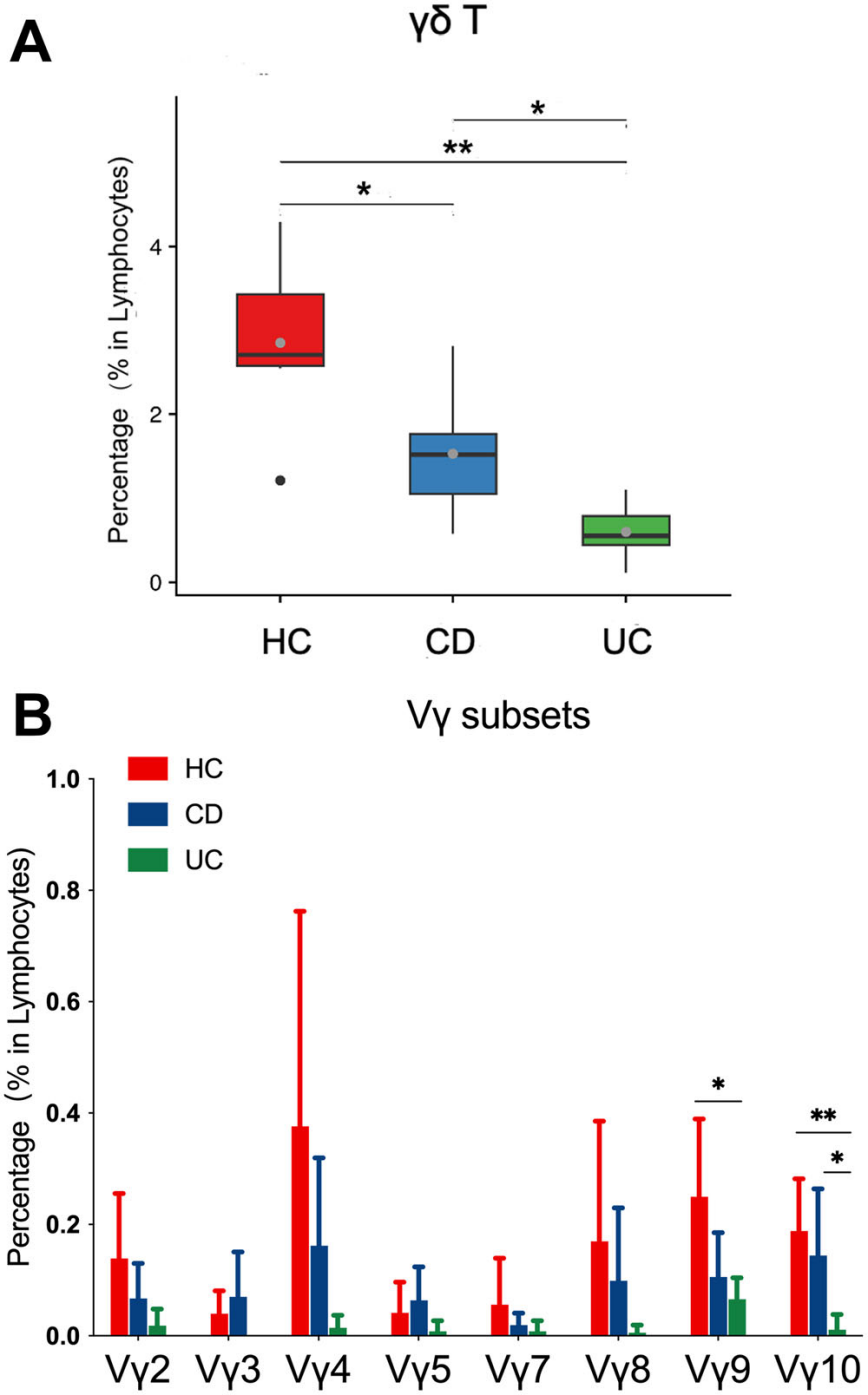
ScRNA‐seq analysis of intestinal γδ T cells and their subsets in CD and UC patients. (A) Pooled scRNA‐seq data identified the percentage of γδ T cells in lymphocytes among HCs, CD patients, and UC patients. (B) Pooled scRNA‐seq data identified the percentages of Vγ subsets in lymphocytes among HCs, CD patients, and UC patients. Healthy control samples (HC, *n* = 6), CD samples (CD, *n* = 6), and UC samples (UC, *n* = 6). Data are mean ± SD. *p < 0.05; **p < 0.01; ***p < 0.001; ****p < 0.0001.

We analyzed the existing single cell RNA‐seq data (GSE214695) from IBD cohorts. First, we analyzed the percentages of intestinal γδ T cells among the three groups, and found that the percentages of intestinal γδT cells in CD and UC groups decreased significantly compared with HCs (Figure 6A). Then we subdivided the intestinal γδT cells of the three groups into different Vγ subsets. To further explore the immune characteristics of different Vγ subsets in CD and UC patients, we analyzed the percentages of each Vγ subsets among the three groups and the expressions of functional molecules in different Vγ subsets among the three groups. The results showed that compared with HCs, the subsets of Vγ 2, Vγ 4, Vγ 7, Vγ 8, Vγ 9, and Vγ 10 in CD group were in a downward trend, while the subsets of Vγ 3 and Vγ 5 were in an upward trend. Compared with HCs, all the Vγ subsets in UC group were in a downward trend, and the downward trend of Vγ 9 and Vγ 10 was statistically significant. Notably, compared with HC and CD groups, Vγ 3 subsets were absent in UC group (Figure 6B). In addition, we found that the expressions of functional molecules (CXCR3, HLA‐DR, PD‐1, Perforin, and Granzyme B) in different Vγ subsets among the three groups were not consistent, which together constitute the overall expression of functional molecules in γδ T cells. The results were included in the Supporting Information S1: Figure A2.

### 3.7 ROC Curve Analysis of Intestinal γδ T Cells in CD and UC Patients

**Figure 7:**
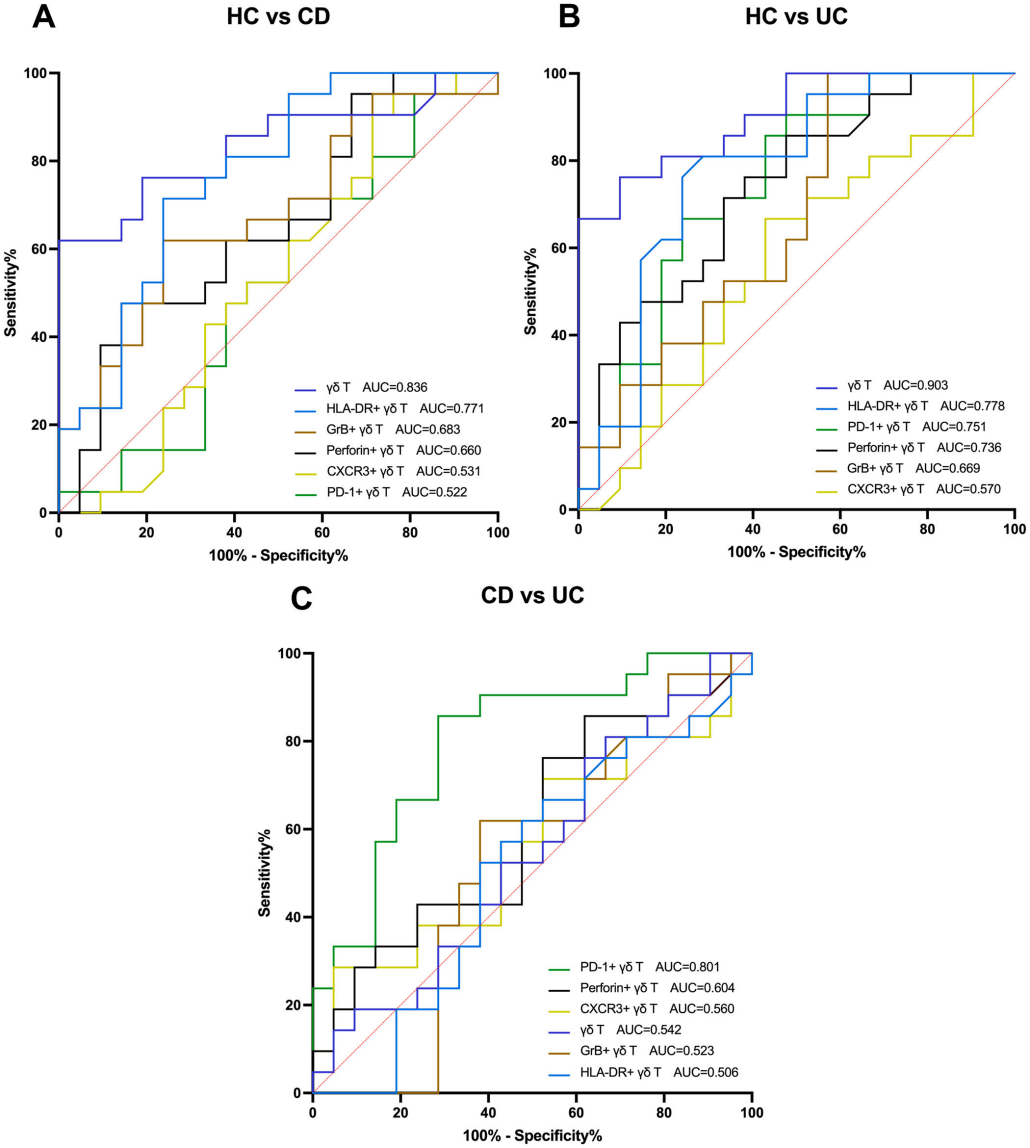
ROC curve analysis of intestinal γδ T cells in CD and UC patients. (A) To distinguish between HC and CD. γδ T cell ratio (AUC, 0.836) and HLA‐DR^+^ γδ T cell ratio (AUC, 0.771) exhibited good diagnostic values for CD. (B) To distinguish between HC and UC. γδ T cell ratio (AUC, 0.903), HLA‐DR^+^ γδ T cell ratio (AUC, 0.778), PD‐1^+^ γδ T cell ratio (AUC, 0.751), and Perforin^+^ γδ T cell ratio (AUC, 0.736) exhibited good diagnostic values for UC. (C) To distinguish CD from UC. PD‐1^+^ γδ T cell ratio (AUC, 0.801) showed a good value to distinguish CD from UC. Other parameters were not statistically significant. AUC is an area under the curve.

We utilized parameters that exhibited distinct expression levels in intestinal γδT cells of healthy subjects, CD patients, and UC patients to diagnose CD or UC, and distinguish CD from UC. Based on ROC curve analysis, we found that γδT cell ratio (AUC, 0.836) and HLA‐DR^+^ γδT cell ratio (AUC, 0.771) exhibited good diagnostic values for CD, with the specificity of 100% and 76.19% and sensitivity of 61.9% and 71.43%, respectively (Figure 7A). In addition, γδT cell ratio (AUC, 0.903), HLA‐DR^+^ γδT cell ratio (AUC, 0.778), PD‐1^+^ γδT cell ratio (AUC, 0.751), and Perforin^+^ γδT cell ratio (AUC, 0.736) exhibited good diagnostic values for UC, with the specificity of 90.48%, 71.43%, 76.19% and 66.67% and sensitivity of 76.19%, 80.95%, 66.67% and 71.43%, respectively (Figure 7B). Furthermore, PD‐1^+^ γδT cell ratio (AUC, 0.801) showed a good value to distinguish CD from UC with the specificity of 71.43% and sensitivity of 85.71% (Figure 7C). Meanwhile, other parameters were not statistically significant (Detailed data are listed in Tables 2, 3, 4).

**Table 2:** Receiver operation characteristic curve parameters for CD diagnosis.

Parameters	AUC	*p*	95% CI	Cut‐off value	Jordan index	Sensitivity	Specificity
γδ T	0.836	0.0002	0.7090–0.9622	< 3.390	0.619	61.9%	100%
HLA‐DR^+^ γδ T	0.771	0.0026	0.6280–0.9139	> 25.20	0.4762	71.43%	76.19%

*Note:* γδ T and HLA‐DR^+^ γδ T refer to the ability of γδ T cell ratio and HLA‐DR^+^ γδ T cell ratio to distinguish between healthy controls and active CD cases.

Abbreviations: AUC, area under the curve; 95% CI, 95% confidence interval.

**Table 3:** Receiver operation characteristic curve parameters for UC diagnosis.

Parameters	AUC	*p*	95% CI	Cut‐off value	Jordan index	Sensitivity	Specificity
γδ T	0.903	< 0.0001	0.8142–0.9908	< 4.265	0.6667	76.19%	90.48%
HLA‐DR^+^ γδ T	0.778	0.0021	0.6317–0.9239	> 23.80	0.5238	80.95%	71.43%
PD‐1^+^ γδ T	0.751	0.0054	0.6016–0.8996	> 86.30	0.4286	66.67%	76.19%
Perforin^+^ γδ T	0.736	0.0089	0.5849–0.8868	< 9.240	0.381	71.43%	66.67%

*Note:* γδ T, HLA‐DR^+^ γδ T, PD‐1^+^ γδ T, and Perforin^+^ γδ T count refer to the ability of γδ T cell ratio, HLA‐DR^+^ γδ T cell ratio, PD‐1^+^ γδ T cell ratio, and Perforin^+^ γδ T cell ratio to distinguish between healthy controls and UC cases.

Abbreviations: AUC, area under the curve; 95% CI, 95% confidence interval.

**Table 4:** Receiver operation characteristic curve parameters to distinguish CD from UC.

Parameters	AUC	*p*	95% CI	Cut‐off value	Jordan index	Sensitivity	Specificity
PD‐1^+^ γδ T	0.801	0.0009	0.6648–0.9361	> 82.65	0.5714	85.71%	71.43%

*Note:* PD‐1^+^ γδ T refer to the ability of PD‐1^+^ γδ T cell ratio to distinguish between CD and UC cases.

Abbreviations: AUC, area under the curve; 95% CI, 95% confidence interval.

## 4 Discussion

Previous studies have shown that the abnormal intestinal inflammatory response in IBD patients was related to the dysregulation of the intestinal immune environment. Most studies over the past few decades have focused on the role of conventional T cells in IBD [18]. Recent studies confirm the significance of γδ T cells in the pathogenesis of IBD. It is unclear how intestinal γδ T cells from CD and UC patients differ in immunological features and their association with disease activity. Therefore, we conducted this study and obtained meaningful results.

First, we assessed the distribution of intestinal γδ T cells in CD and UC patients. The results showed that the frequency of intestinal γδ T cells in CD and UC patients was significantly decreased compared to the HCs, which was also confirmed by our single cell data analysis. The decrease in γδ T cell frequency in the intestinal mucosa of CD and UC patients may have numerous causes: 1. The patient's intestinal mucosa has a relative decrease in γδ T cell frequency due to αβ T cell expansion. 2. The altered migratory capacity of intestinal γδ T cells, resulting in the migration of intestinal γδ T cells to other sites, thereby reducing the frequency of intestinal γδT cells. 3. Intestinal γδ T cell frequency decreased due to increased cell mortality and decreased proliferation. We found no significant difference in intestinal αβ T cells in the three groups of subjects. Thus, we learned that the decreased frequency of intestinal γδ T cells in the patients was not caused by the expansion of αβ T cells. To further investigate the reason for the decreased frequency of intestinal γδ T cells, we examined the migratory capacity of intestinal γδ T cells. Under inflammatory conditions, CXCR3 on human γδ T cells binds to chemokines generated by nearby cells, recruiting CXCR3+ γδ T cells to inflammatory lesions [19, 20]. However, by detecting the CXCR3 expression on intestinal γδ T cells in the enrolled cohorts, we found that the CXCR3 expression on intestinal γδ T cells had no significant differences among the three groups. Thereby, we learned that the decreased frequency of intestinal γδ T cells in the patients was also not caused by the altered migratory capacity of intestinal γδ T cells. Therefore, we hypothesized that decreased intestinal γδ T cell frequency in CD and UC patients' intestinal mucosa may be due to increased cell mortality and reduced cell proliferation. Regarding this phenomenon of γδ T cell reduction in CD and UC patients' intestinal mucosa, we speculated that it might be related to immune disorders in the intestinal mucosa of patients. There was substantial evidence for the protective role of γδ T cells in intestinal inflammation in several murine models [21, 22]. One study found that reducing γδ T cells increased the inflammation severity and even the mortality of rats with colitis [23]. Through research, we further found that the frequency of intestinal γδ T cells did not decrease in mildly active CD patients but decreased in moderately active CD patients with disease activity progression. Furthermore, intestinal γδ T cell frequency considerably decreased in moderate and severe UC patients. Thus, decreased intestinal γδ T cells in CD and UC patients identified might be associated with disease activity. Contrary to our findings, McVay et al. found that increased numbers of gamma delta T cells localized in areas of inflammation and tissue injury were found in the majority of patients, irrespective of the type of IBD present [24]. Through comparison, we found that McVay et al. selected specimen type was surgical specimens. While we thought that there were many factors interfering with the surgical specimens. Therefore, we firmly choose biopsy specimens, and require patients in the group not to undergo intestinal surgery in the past year. Therefore, the results are inconsistent due to different types of research samples. In addition, Giacomelli et al. found the increase of circulating gamma delta T lymphocytes in the peripheral blood of patients affected by active inflammatory bowel disease [25]. The change trend of γδT cells in peripheral blood discovered by Giacomelli et al. is just the opposite to that of γδT cells in intestine discovered by our research. Whether there is a certain relationship between the two parts of γδT cells needs further discussion in the future.

Next, we explored the immune function of intestinal γδ T cells in CD and UC patients. It was found that the intestinal γδ T cells of CD patients showed elevated HLA‐DR expression but no differences in PD‐1, Perforin, or Granzyme B expressions compared to HCs. This indicated that intestinal γδ T cells of CD patients were in a highly activated state, and their cytotoxicity was unimpaired. We further found that the inconsistent intestinal inflammatory environment of CD patients with different disease activity stages might make the intestinal γδ T cells in an activated, inhibited or resting state. The intestinal γδ T cells in CD patients with mild activity could be activated and play an active anti‐inflammatory role under the relatively mild stimulation of inflammation. As disease activity increased, intestinal γδ T cells in CD patients with moderate activity failed to activate to play an anti‐inflammatory role under stronger and longer inflammatory stimulation.

Furthermore, we found that the HLA‐DR expression on intestinal γδ T cells in UC patients was increased compared to HCs, indicating that intestinal γδ T cells were in a highly activated state. Still, we found that compared to HCs, increased PD‐1 expression and decreased Perforin and Granzyme B expressions were shown in intestinal γδ T cells of UC patients, indicating that intestinal γδ T cells might also be in an immunosuppressed state, with significantly impaired cytotoxicity. We inferred that it might be because the UC cohorts contained patients with variable disease activity, and these patients had different γδ T cell characteristics, which may explain the seemingly implausible results. Subsequently, we studied the immune function of intestinal γδ T cells in UC patients with varying degrees of disease activity. We found that intestinal γδ T cells were activated with unimpaired cytotoxicity of moderately active UC patients. Therefore, we speculated that intestinal γδ T cells in this state could positively affect the immune response. However, as disease activity increased, intestinal γδ T cells were suppressed with impaired cytotoxicity of UC patients with severe activity. Hence, we speculated that intestinal γδ T cells in this state could not maintain their positive immune response.

Previous studies reported that γδT cells can be subdivided into different Vγ subsets, which may play different roles [26]. Therefore, we further analyzed the immune characteristics of Vγ subsets in IBD patients by analyzing the existing scRNA‐seq data (GSE214695) from IBD cohorts (HC, CD, and UC) [17]. We found that the percentages of each Vγ subsets and the expression of its functional molecules (CXCR3, HLA‐DR, PD‐1, Perforin, and Granzyme B) were different among the three groups, which together constitute the percentages and immune characteristics of γδ T cells. Different Vγ subsets in CD or UC patients present different immune characteristics, which may explain the differences in immune characteristics of γδT cells in CD or UC patients with different disease activity.

These findings have allowed us to understand the similarities and differences in immune characteristics of intestinal γδ T cells among healthy individuals, CD patients, and UC patients. Therefore, we further analyzed the ROC curves and found that γδ T cell ratio (AUC, 0.836; Sp, 100%; Se, 61.9%) and HLA‐DR^+^ γδ T cell ratio (AUC, 0.771; Sp, 76.19%; Se, 71.43%) exhibited good values for assisting diagnosis of CD. Furthermore, γδ T cell ratio (AUC, 0.903; Sp, 90.48%; Se, 76.19%), HLA‐DR^+^ γδ T cell ratio (AUC, 0.778; Sp, 71.43%; Se, 80.95%), PD‐1^+^ γδ T cell ratio (AUC, 0.751; Sp, 76.19%; Se, 66.67%), and Perforin^+^ γδ T cell ratio (AUC, 0.736; Sp, 66.67%; Se, 71.43%) exhibited good values for assisting diagnosis of UC. In addition, we found that PD‐1^+^ γδ T cell ratio (AUC, 0.801; Sp, 71.43%; Se, 85.71%) was a valuable indicator to help distinguish CD from UC. These insights offered valuable theoretical foundations for the precise diagnosis of CD or UC and the distinction between CD and UC. At present, clinicians can't diagnose patients with CD and UC quickly and accurately according to the existing laboratory indicators. Although pathological diagnosis is the gold standard, the diagnosis time is too long (about 1 week). We still need to find accurate, convenient and fast diagnosis methods to win critical treatment time for patients. In this study, we don't need a separate invasion to obtain samples, but we can take another small biopsy tissue during the necessary pathological biopsy. Then, the valuable biomarkers in γδ T cells can be detected by flow cytometry, and the results can be obtained in about 6 h to assist clinicians in rapid diagnosis.

## 5 Conclusions

This study found that the immune characteristics of intestinal γδ T cells in CD and UC patients showed certain similarities and differences and were closely related to disease activity. With increasing disease activity, intestinal γδ T cells of CD and UC patients decreased. The reason was not related to the growth of intestinal αβT cells or their migratory capacity but rather to the reduction of γδ T cells due to increased cell death and decreased proliferation. Moreover, with the increased disease activity, the decreased intestinal γδ T cells in CD and UC patients could not activate in response to the inflammatory stimuli. The differences were that with the increased disease activity, the cytotoxicity of intestinal γδ T cells in CD patients was always normal. In contrast, the cytotoxicity of intestinal γδ T cells in UC patients was inhibited. In addition, different Vγ subsets in CD or UC patients show different immune characteristics, which may explain the differences in immune characteristics of γδT cells in CD or UC patients with different disease activity. Furthermore, γδ T cell and HLA‐DR^+^ γδT cell ratio were identified as good indicators in the diagnosis of CD. The ratios of γδ T cell, HLA‐DR^+^ γδ T cell, PD‐1^+^ γδ T cell, and Perforin^+^ γδ T cell exhibited good values for assisting diagnosis of UC. PD‐1^+^ γδ T cell ratio was a valuable indicator to help distinguish CD from UC. These meaningful indicators might become potential immune biomarkers. These findings provide new perspectives and theoretical bases for future CD and UC patients' immunotherapy.

## Author Contributions

**Yujie Jiang:** investigation, methodology, writing – original draft. **linna Ye:** investigation, resources. **Caixia Sheng:** validation. **Jia Zhu:** visualization. **Jiaqi Xu:** funding acquisition, writing – review and editing. **Xiaoqing Cheng:** formal analysis. **Guoxiang Fu:** project administration. **Zhinong Jiang:** funding acquisition, project administration, supervision, writing – review and editing.

## Ethics Statement

The study was conducted in accordance with the Declaration of Helsinki and approved by the ethics committee of Sir Run Run Shaw Hospital Affiliated with Zhejiang University School of Medicine (number: 2022‐0293).

## Consent

Informed consent was obtained from all subjects involved in the study. Written informed consent has been obtained from the patient(s) to publish this paper.

## Conflicts of Interest

The authors declare no conflict of interest.

## Supporting information

FigureA1. **The Fluorescence Minus One staining results.** To account for the continuous expression pattern of fluorescent markers on the partial antibodies (CXCR3, HLA‐DR, PD‐1, Perforin, and Granzyme B), we employed Fluorescence Minus One (FMO) staining. FigureA2. **Pooled scRNA‐seq data identified the expression of functional molecules (CXCR3, HLA‐DR, PD‐1, Perforin, and Granzyme B) in different Vγ subsets among HCs, CD patients, and UC patients**. Healthy control samples (HC, n = 6), CD samples (CD, n = 6), and UC samples (UC, n = 6). Data are mean ± SD. *, p < 0.05; **, p < 0.01; ***, p < 0.001; ****, p < 0.0001.

## Data Availability

The data underlying this article are available in the article and its online supplementary information.
