# Supplementary material for: Similarities and Differences in the Immune Characteristics of Intestinal Gamma Delta T Cells From Patients With Crohn's Disease and Ulcerative Colitis and Their Correlation With Disease Activity
Source: Immun Inflamm Dis. 2025 Oct 15;13(10):e70273. doi: 10.1002/iid3.70273 (PMC12521879; doi:10.1002/iid3.70273)
Supplement: Supplementary file 1 — FigureA1. The Fluorescence Minus One staining results. To account for the continuous expression pattern of fluorescent markers on the partial antibodies (CXCR3, HLA‐DR, PD‐1, Perforin, and Granzyme B), we employed Fluorescence Minus One (FMO) staining. FigureA2. Pooled scRNA‐seq data identified the expression of functional molecules (CXCR3, HLA‐DR, PD‐1, Perforin, and Granzyme B) in different Vγ subsets among HCs, CD patients, and UC patients. Healthy control samples (HC, n = 6), CD samples (CD, n = 6), and UC samples (UC, n = 6). Data are mean ± SD. *, p < 0.05; **, p < 0.01; ***, p < 0.001; ****, p < 0.0001. [file IID3-13-e70273-s001.docx]

**Supplementary material:**


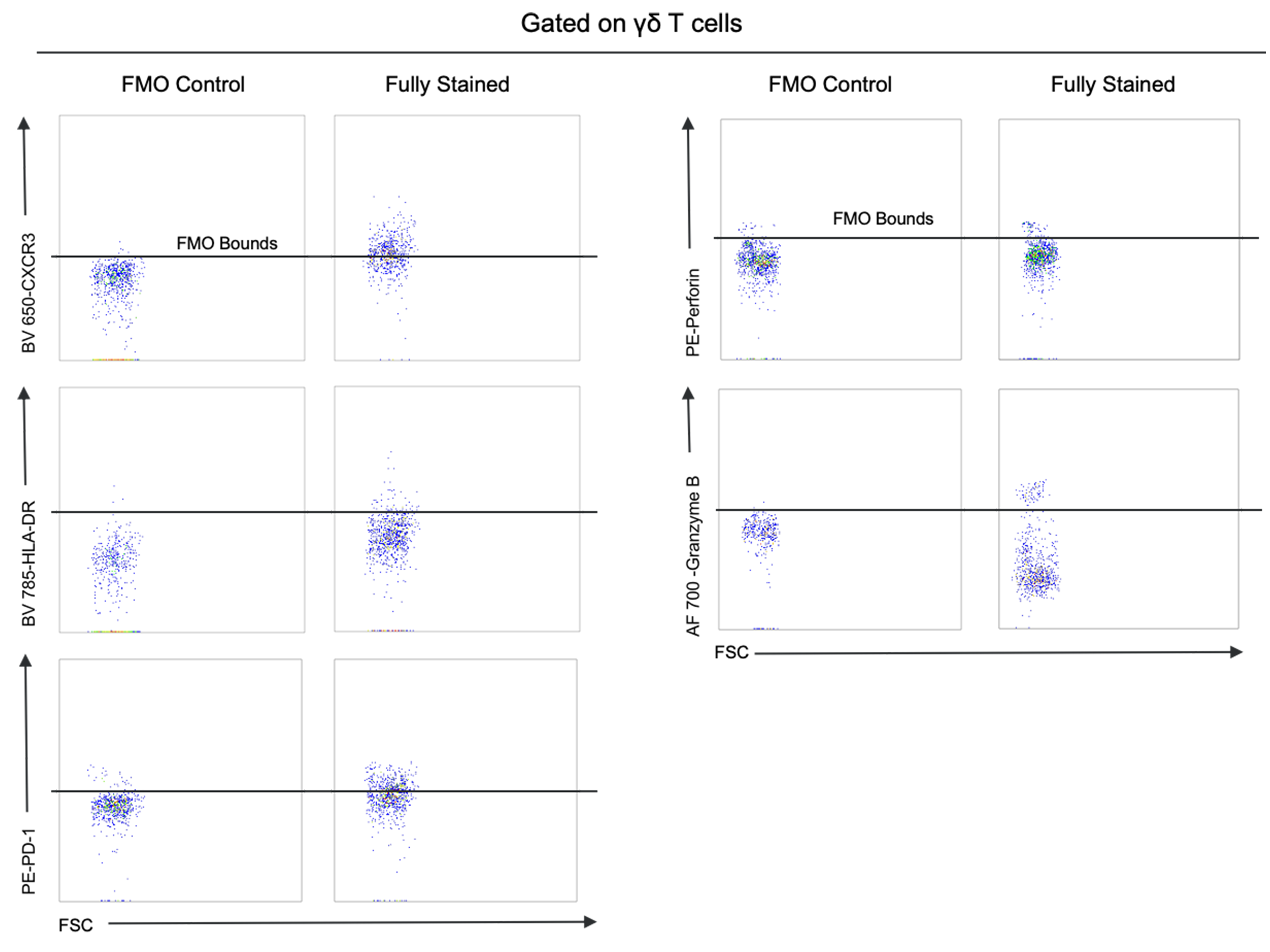


**Fig.A1. The Fluorescence Minus One staining results.** To account for the continuous expression pattern of fluorescent markers on the partial antibodies (CXCR3, HLA-DR, PD-1, Perforin, and Granzyme B), we employed Fluorescence Minus One (FMO) staining.


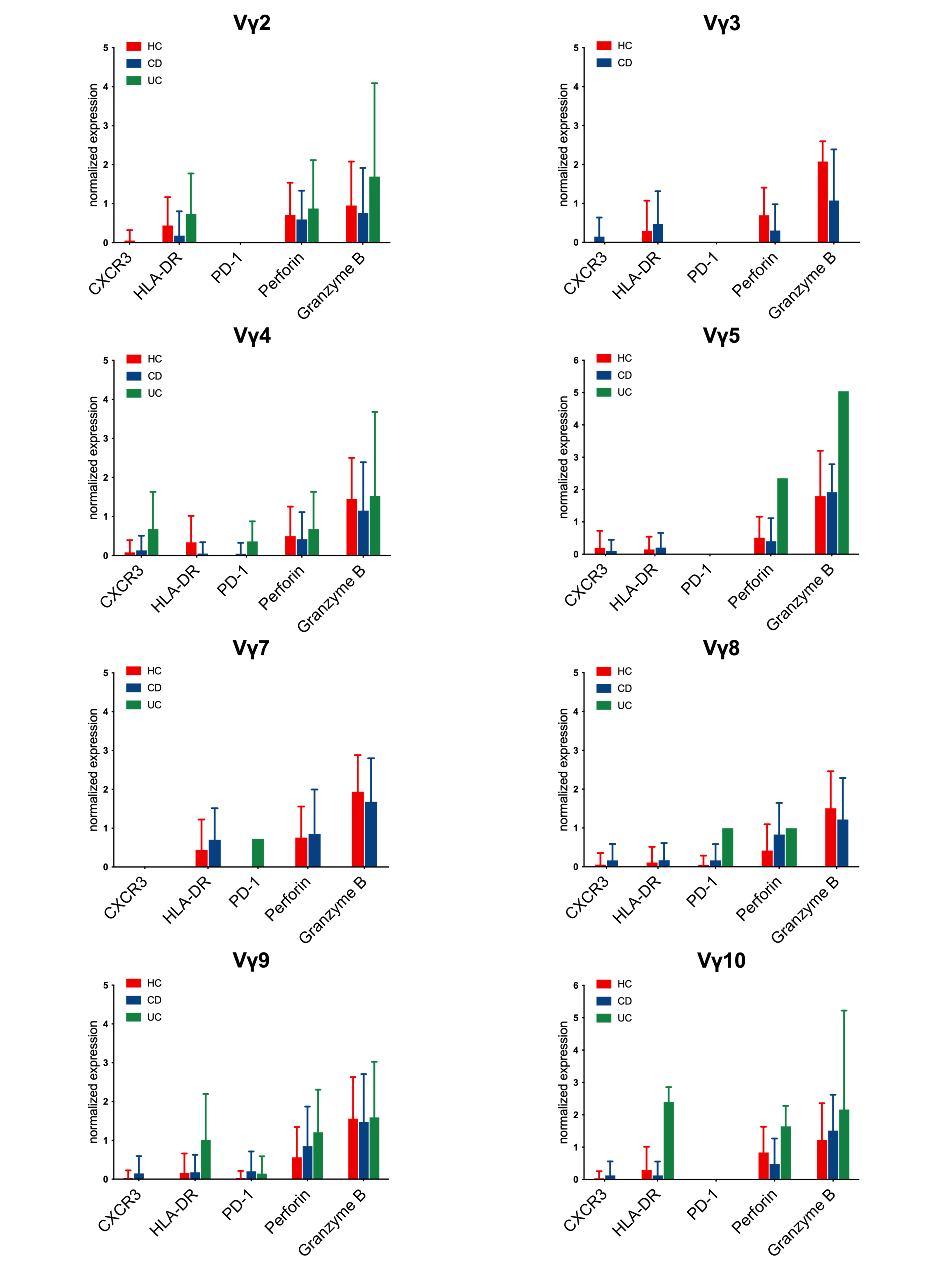


**Fig.A2. Pooled scRNA-seq data identified the expression of functional molecules (CXCR3, HLA-DR, PD-1, Perforin, and Granzyme B) in different Vγ subsets among HCs, CD patients, and UC patients.** Healthy control samples (HC, n = 6) , CD samples (CD, n = 6) , and UC samples (UC, n = 6). Data are mean ± SD. *, p < 0.05; **, p < 0.01; ***, p < 0.001; ****, p < 0.0001.
